# Chronodisruption of the acute inflammatory response by night lighting in rats

**DOI:** 10.1038/s41598-023-41266-3

**Published:** 2023-08-29

**Authors:** Viera Jerigova, Michal Zeman, Monika Okuliarova

**Affiliations:** Department of Animal Physiology and Ethology, Faculty of Natural Sciences, Comenius University, Ilkovicova 6, 842 15 Bratislava, Slovakia

**Keywords:** Acute inflammation, Risk factors, Kidney

## Abstract

Daily oscillations are present in many aspects of the immune system, including responsiveness to infections, allowing temporal alignment of defence mechanisms with the external environment. Our study addresses whether compromised circadian timing function by dim artificial light at night (ALAN) impacts the time dependency of the acute inflammatory response in a rat model of lipopolysaccharide (LPS)-induced inflammation. After 2 weeks of exposure to low-intensity ALAN (~2 lx) or a standard light/dark cycle, male rats were challenged with LPS during either the day or the night. Dim ALAN attenuated the anorectic response when rats were stimulated during their early light phase. Next, ALAN suppressed daily variability in inflammatory changes in blood leukocyte numbers and increased the daytime sensitivity of neutrophils to the priming effects of LPS on oxidative burst. An altered renal inflammatory response in ALAN-exposed rats was manifested by stimulated T-cell infiltration into the kidney upon night-time LPS injection and the modified rhythmic response of genes involved in inflammatory pathways. Moreover, ALAN disturbed steady-state oscillations of the renal molecular clock and eliminated the inflammatory responsiveness of *Rev-erbα*. Altogether, dim ALAN impaired time-of-day-dependent sensitivity of inflammatory processes, pointing out a causal mechanism between light pollution and negative health effects.

## Introduction

Innate immune mechanisms are activated as the first line of defence upon infection or tissue injury. The acute inflammatory response, a part of innate immunity, involves behavioural and physiological changes that are mediated by a wide spectrum of immune chemical regulators (e.g., cytokines, acute phase proteins and hormones) and immune cells^1^. The course of the inflammatory response is documented by dynamic changes in the number of circulating white blood cells (WBCs), which reflects the rate of their recruitment into the tissues and their formation and supply from the bone marrow^2^. The magnitude of the inflammatory response must be tightly regulated to ensure elimination of the triggering stimulus and simultaneously to minimize collateral damage of host tissues^3^.

The time of day at which the infection attacks the host has been shown to significantly affect the strength of the inflammatory response^4, 5^. This daily variability is expected to be driven by circadian rhythms, which have been identified in most immune parameters, including WBC trafficking^6^. Endogenous circadian rhythms are generated by circadian clocks that have evolved to maintain temporal synchrony of individual body systems and processes with each other, as well as with periodic fluctuations in the environment^7^. The light/dark (L/D) cycle is the most important environmental cycle entraining the mammalian circadian system. Light information first enters the central clock in the suprachiasmatic nuclei (SCN) of the hypothalamus, which in turn transmits a synchronized signal to the peripheral cell-autonomous oscillators via rhythms in neural and humoral pathways^8^. In the modern world, marked by widespread light pollution and a lifestyle independent of the solar day, optimal timekeeping function of the circadian system is extensively challenged by exposure to artificial light at night (ALAN)^9, 10^. Experimental studies in rats have demonstrated that dim ALAN (≤ 5 lx) compromises circadian organisation by dampening the rhythmicity of the central clock and suppressing or abolishing the rhythmic pattern of specific metabolic genes, hormones and behaviour^11, 12^.

Disruption of circadian timing function can also impact daily rhythms in the immune system and in turn disturb host defence mechanisms^13^. A recent study showed that rats exposed to dim ALAN exhibited impaired daily variation of the main leukocyte subsets in the blood, which was associated with reduced blood monocyte counts and disturbed immune homeostasis in the kidney^14^. Dim ALAN has also been reported to alter the responsiveness of the immune system under experimentally induced acute inflammation. Specifically, an excessive inflammatory response was found in mice^15^, whereas diminished immune responses were observed in Siberian hamsters exposed to dim ALAN^16^. Such variable immune responsiveness under ALAN conditions can result from disruption of circadian rhythms in the immune system. However, data examining the effects of ALAN on the immune response induced at more than one time point over 24 h are missing.

Therefore, here we aimed to evaluate diverse aspects of the acute inflammatory response in a time-of-day-dependent manner and whether dim ALAN can disrupt this daily rhythmicity through the effects on leukocyte trafficking between the blood and tissues. We focused on the immune state in the kidney and renal immune cells, which participate profoundly in tissue homeostasis and shape the course of the disease response^17^. Moreover, kidney inflammation is an important player in renal kidney injury and chronic kidney disease, while the progression of these pathologies has been shown to accelerate by circadian disruption^18^. In our study, we used a model of lipopolysaccharide (LPS)-induced inflammation in rats that were exposed to dim ALAN (~2 lx) for 2 weeks.

## Materials and methods

### Animals

Male Wistar rats (282 ± 4 g) were obtained from the breeding station of the Institute of Experimental Pharmacology and Toxicology, Slovak Academy of Sciences (Dobrá Voda, Slovak Republic). The animals were housed in plastic cages in groups of three to four rats at an ambient temperature of 21.5 ± 1.3 °C and humidity of 55–65%. A standard pelleted diet and water were provided *ad libitum*. During the 2-week acclimation period, all rats were adapted to the 12/12 L/D cycle with lights on at 6:00 h (designed as Zeitgeber time 0, ZT0). Broad-spectrum white light with an illumination of 150–200 lx and a colour temperature of 2900 K was used during the daytime. Rats were assigned to either the control group (CTRL, n = 28) with the standard lighting regime described above or to the experimental group (ALAN, n = 30), which was exposed to low-illuminance levels of 2 lx during the entire dark phase^11^. Dim illuminance was provided by a LED light strip with colour temperature of 3,000 K. Illuminance and colour temperature were measured at the level of the animal cages using an illuminance spectrophotometer CL-500A (Konica Minolta Sensing Europe BV, Bremen, Germany)^12^.

The experimental procedures followed the ARRIVE guidelines and they were approved by the Ethical Committee for the Care and Use of Laboratory Animals at the Comenius University in Bratislava, Slovak Republic and the State Veterinary Authority of the Slovak Republic (Ro-1648/19-221/3). All methods were performed in accordance with the relevant guidelines and regulations.

### Experimental design

**Figure 1:**
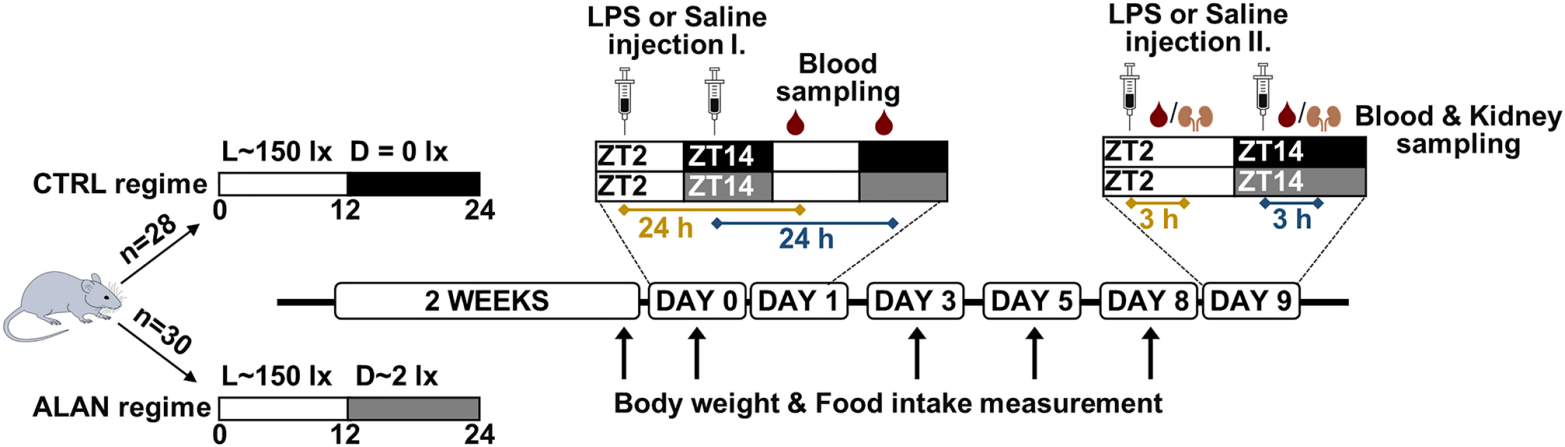
Experimental design. Rats were exposed to either the control light/dark (L/D) regime (CTRL, n=28) or dim artificial light (~2 lx) at night (ALAN, n = 30) for 2 weeks, Thereafter, on day 0, one half of each group received saline or lipopolysaccharide (LPS) injection I. at ZT2 and the other half was injected at ZT14 (ZT0 = lights on). Blood samples were collected 24 h post-injection. Body weight and food intake were monitored for the next 8 days. On day 9, rats received saline or LPS injection II. at the same times of day as the first one. Animals were sacrificed 3 h later to collect blood and kidney samples.

After 2 weeks, CTRL and ALAN-exposed rats were injected intraperitoneally (i.p.) with either sterile saline or LPS from *Escherichia coli* (serotype 0111:B4; Sigma-Aldrich, Saint Louis, MO, USA) at a dose of 1 mg/kg body weight. One half of each group was challenged at the beginning of the light phase (ZT2) and the other half at the beginning of the dark/dim light phase (ZT14). Then, 24 h post-saline or LPS injection, rats were anaesthetised by isoflurane inhalation, and blood was collected from a lateral tail vein into tubes with either ethylenediaminetetraacetic acid (EDTA, for immunophenotypic analysis) or heparin (for analysis of the oxidative burst of neutrophils). The LPS injection and blood sampling were performed under a dim red light during the dark phase. Body weight and food intake were recorded before stimulation and on days 3, 5 and 8 post-immune challenge. To analyse acute inflammatory changes, rats received on day 9 another i.p. injection of saline or LPS (at the same times of day as the first one) and were sacrificed 3 h later under brief isoflurane anaesthesia. Blood and tissue samples were collected. Serum and EDTA plasma were separated by centrifugation at 2500 g, 15 min, 4 °C and used for measurements of hormones and cytokines by radioimmunoassay (RIA) and enzyme-linked immunosorbent assay (ELISA). The left kidney was dissected and cut into three pieces. The middle part was processed for immunostaining, and both ends were immediately frozen in liquid nitrogen and used for protein and gene expression analyses by Western blot and real-time PCR (qPCR), respectively. Samples were stored at –76 °C. Schematic experimental design is illustrated in Figure 1.

### Immunophenotypic analysis

To examine the numbers of specific WBC populations, aliquots of blood (50 µL) were stained with three panels of fluorescence-labelled antibodies and incubated for 30 min at 4 °C in the dark. Red blood cells were lysed using ammonium chloride–potassium buffer for 5 min. After washing, WBCs were dissolved in 0.3 mL of phosphate-buffered saline (PBS) supplemented with 0.5% bovine serum albumin (BSA) and 0.1% sodium azide and analysed on a BD Accuri C6 cytometer (BD Bioscience, San Diego, CA, USA). The list of fluorochrome-conjugated monoclonal antibodies is in Supplementary Table S1. Leukocyte subsets were identified based on specific surface markers in the gate for total leukocytes (CD45^+^): T cells (CD3^+^), helper T cells (CD3^+^CD4^+^), cytotoxic T cells (CD3^+^CD8a^+^), B cells (CD45RA^+^), NK cells (CD3^−^CD161a^+^), neutrophils and monocytes (HIS48^+^ and side scatter gating)^14^. Reciprocal expression of CD43 and HIS48 markers was used to identify classical (CD43^lo^HIS48^hi^) or non-classical monocytes (CD43^hi^HIS48^lo^)^14, 19^. All cytometric data were evaluated using FlowJo software (TreeStar, Ashland, OR, USA).

### Oxidative burst of neutrophils

The oxidative burst of neutrophils was measured in whole heparinised blood diluted with PBS using a flow cytometry-based assay with 2′,7′-dichlorodihydrofluorescein diacetate (H_2_-DCF-DA; Sigma-Aldrich)^14^. Within the cell, H_2_-DCF-DA is converted to fluorescent 2′,7′-dichlorofluorescein (DCF), and this fluorescence is directly proportional to the formation of reactive oxygen species (ROS)^20^. The oxidative burst of the neutrophils was expressed as the fold increase in the median DCF fluorescence of the phorbol-12-myristate-13-acetate-stimulated aliquots over the non-stimulated aliquots.

### Immunofluorescence and immunohistochemistry

The kidney samples were fixed, stored at –76 °C, and immunostained according to the previously published protocol^14^. Briefly, frozen samples were cut into 8-µm-thick sections, which were mounted on adhesive slides. For immunofluorescence, the sections were rehydrated, incubated with 50 mM ammonium chloride for 30 min followed by 0.25% Triton X-100 in PBS for 10 min and blocked with 5% goat serum in PBS for 1 h at room temperature. Then, the sections were stained with the following primary antibodies: mouse anti-rat CD68 (MCA341R, Bio-Rad, Hercules, CA, USA; diluted 1:100) to identify macrophages or rabbit antibody against myeloperoxidase (MPO) (ab9535, Abcam, Cambridge, UK; diluted 1:25) to identify neutrophils. After overnight incubation at 4 °C in the dark, the sections were stained with the following secondary antibodies: anti-mouse Alexa Fluor 660 (A-21055, Thermo Fisher Scientific, Waltham, MA, USA; diluted 1:750) and anti-rabbit Alexa Fluor 488 (A-11008, Thermo Fisher Scientific; diluted 1:500) for 1 h at room temperature. Cell nuclei were counterstained with DAPI (Roche, Indianapolis, IN, USA; diluted 1:10,000). Positive signals were analysed using a Zeiss Axioscope fluorescence microscope (Carl Zeiss, Oberkochen, Germany).

Immunohistochemistry was used to quantify T cells in the renal cortex. First, the rehydrated sections were incubated with Hydrogen Peroxide Blocking Reagent (Abcam) for 10 min to eliminate endogenous peroxidase activity. Next, the sections were treated with 5% goat serum for 1 h at room temperature and then with the Avidin/Biotin Blocking Kit (Vector Laboratories, Burlingame, CA, USA). The sections were stained with rabbit anti-CD3 primary antibody (ab16669, Abcam; diluted 1:300) overnight at 4 °C and biotinylated anti-rabbit IgG secondary antibody (BA-1000, Vector Laboratories; diluted 1:200) for 1 h at room temperature. The biotinylated signal was detected with Vectastain Elite ABC-HRP Reagent (Vector Laboratories) and diaminobenzidine (DAB Substrate Kit, Vector Laboratories). Finally, cell nuclei were counterstained with Mayer’s haematoxylin (DiaPath, Martinengo, Italy). After staining, the sections were dehydrated in a graded series of ethanol, cleared in xylene and coverslipped with DPX mounting medium (Sigma-Aldrich). Stained sections were imaged using light microscopy.

The CD68^+^, MPO^+^ and CD3^+^ cells were counted in 30 randomly selected images of the renal cortex and averaged from two sections per individual. The cell numbers were calculated per mm^2^.

### Western blot

Samples of the renal cortex were homogenised on ice in sucrose buffer supplemented with protease inhibitors^14^. The protein concentrations were determined with the BCA protein assay kit (23227, Thermo Fisher Scientific). Total proteins (20–60 µg) were separated with 10%–12% SDS-PAGE and transferred to a 0.45-µm nitrocellulose membrane. Membranes were blocked with either 5% non-fat dry milk or 5% BSA in Tris-buffered saline containing Tween 20 (TBS-T). Afterwards, the membranes were incubated with rabbit anti-Lipocalin-2 (NGAL) (ab63929, Abcam; diluted 1:1000), rabbit anti-phosphorylated-NF-κB/p65 (Ser536) (ab76302, Abcam; diluted 1:1000) or rabbit anti-NF-κB/p65 (ab16502, Abcam; diluted 1:1000) primary antibodies overnight at 4 °C or with mouse anti-glyceraldehyde-3-phosphate dehydrogenase (GAPDH) antibody (MAB374, Sigma-Aldrich; diluted 1:5000) for 1 h at room temperature. Then the membranes were incubated with the appropriate horseradish peroxidase-conjugated secondary antibodies: anti-mouse (7076, Cell Signaling Technology, Danvers, MA, USA; dilution 1:2000) or anti-rabbit (7074, Cell Signaling Technology; dilution 1:2000) for 1 h at room temperature. Primary and secondary antibodies were diluted in TBS-T containing either 1% non-fat dry milk or 1% BSA. The signals were visualised with Clarity Western ECL substrate (Bio-Rad) using the Vü-C chemiluminescence Imaging System (Pop-Bio Imaging, Cambridge, UK) and quantified with Image Studio Lite Software (LI-COR Biosciences, Lincoln, NE, USA). The amount of target proteins was normalized relative to that of GAPDH.

### RIA and ELISA

Plasma melatonin levels were analysed by RIA as previously described^11^. All samples were measured within a single assay with an intra-assay coefficient of variation of 7.3%. Serum corticosterone (CORT) levels were determined using the rat corticosterone ^125^I RIA kit (RIA-1364, DRG Instruments GmBH, Marburg, Germany), according to the manufacturer’s instructions. ELISA kits for rats were used to measure plasma levels of C-reactive protein (CRP) (88-7501, Thermo Fisher Scientific) and tumour necrosis factor alpha (TNF-α) (ER1393, FineTest, Wuhan Fine Biotech, Wuhan, China).

### RNA isolation and qPCR

Total RNA from the renal cortex was isolated as previously described^14^. For synthesis of complementary DNA, a Maxima cDNA synthesis kit (Thermo Fisher Scientific) was used. Amplification of cDNA was performed with Maxima SYBR Green qPCR Master Mix (Thermo Fisher Scientific) and the CFX Connect real-time PCR detection system (Bio-Rad). The relative expression of the target and reference genes was calculated using a standard curve method. The expression of the target genes was normalized to the expression of the ribosomal protein S29 (*Rps29*). Primer sequences are listed in Supplementary Table S2.

### Statistical analysis

Statistical analysis was performed using GraphPad Prism v.8 (San Diego, CA, USA). Data for body weight and daily food intake were evaluated by two-way repeated measures analysis of variance (ANOVA) with Sidak’s multiple comparisons test. Multiple *t*-tests with Holm-Sidak correction were used to compare the percentage change in body weight between CTRL and ALAN groups. Data for all other variables were analysed by two-way ANOVA with Bonferroni’s multiple comparisons test. Time-of-day dependent differences in LPS response (ZT2 vs. ZT14) were compared within CTRL and ALAN groups with either the Student’s *t*-test or the Mann–Whitney test, depending on the normal distribution. Specific details of the statistics are given in the figure legends. Data are presented as means ± standard error of the mean (SEM).

## Results

### Effects of ALAN on symptoms of LPS-induced sickness

**Figure 2:**
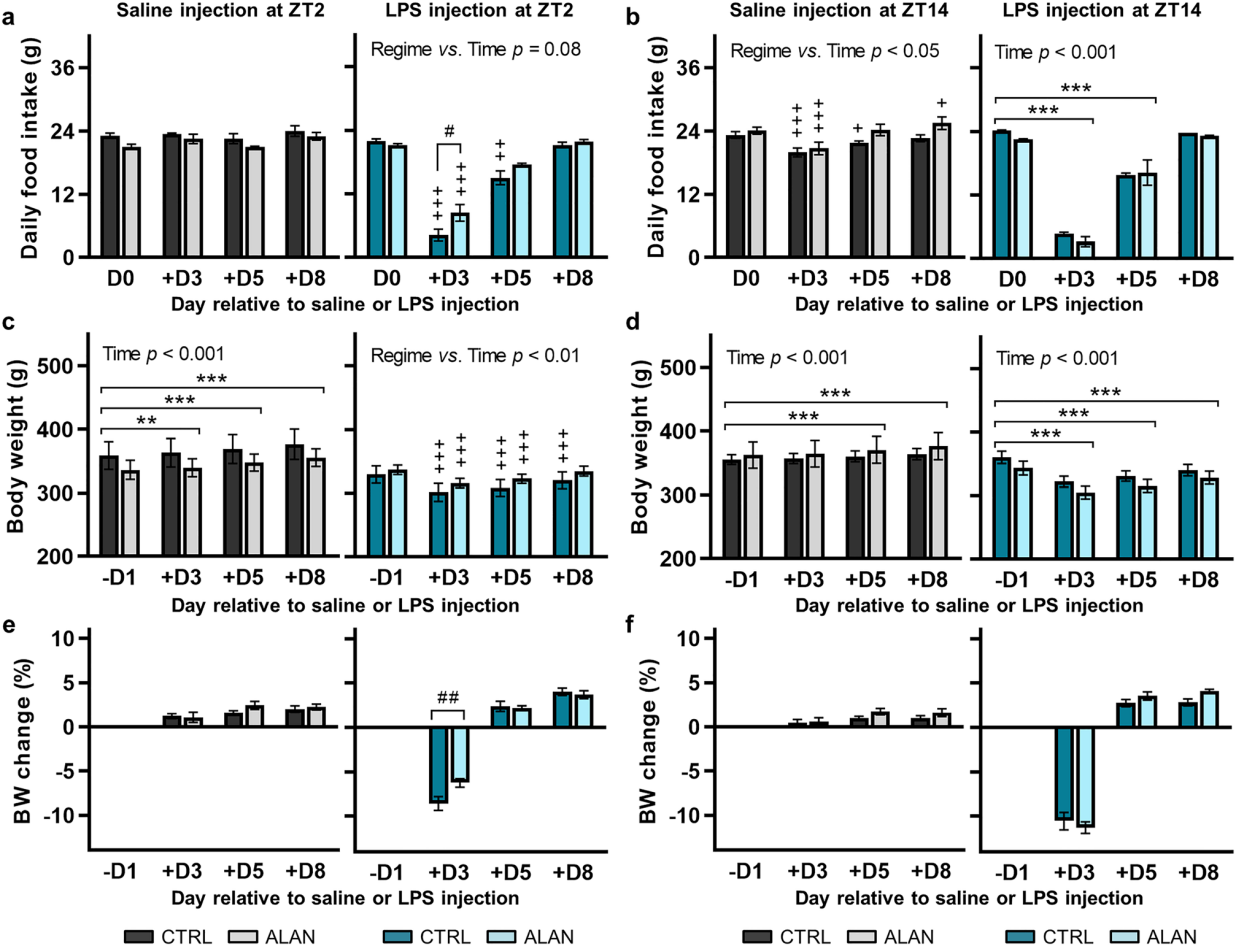
ALAN attenuates the anorectic response to lipopolysaccharide (LPS) during the early light phase. (**a**, **b**) Daily food intake, (**c**, **d**) body weight (BW) and (**e**, **f**) the percentage change in BW after time-of-day-dependent saline or LPS injection in rats exposed to either the control light/dark regime (CTRL) or artificial light at night (ALAN, ~2 lx). Data are shown on day relative to the day of injection (D0) at either Zeitgeber time (ZT) 2 or ZT14. ZT0 = lights on. Bars represent the mean ± SEM (n = 7–8 rats per group). (**a**–**d**) Data for daily food intake and BW were evaluated by two-way repeated measures ANOVA with Sidak’s multiple comparisons test. (**e**, **f**) The percentage change in BW between CTRL and ALAN groups was compared using multiple *t*-tests with Holm-Sidak correction. **p < 0.01 and ***p < 0.001 for comparisons -D1/D0 versus +D3, +D5, +D8 and ^+^*p* < 0.05, ^++^*p* < 0.01 and ^+++^*p* < 0.001 for comparisons -D1/D0 versus +D3, +D5, +D8 within CTRL and ALAN groups in the case of significant interaction. ^#^*p* < 0.05 and ^##^*p* < 0.01 for comparisons CTRL versus ALAN.

We first monitored the effects of ALAN on LPS-induced sickness behaviour in relation to the timing of LPS administration. Control and ALAN-exposed rats did not differ in their daily food intake and body weight prior to LPS administration. Three days after the endotoxin challenge at ZT2, daily food intake was significantly reduced in rats on both the CTRL and the ALAN regime (*p* < 0.001), but ALAN-exposed rats exhibited a smaller decline than controls (*p <* 0.05; Fig. 2a). In CTRL animals, food intake was still lower on day 5 as compared with pre-stimulation levels (*p <* 0.01) and was fully restored 8 days post-LPS injection in both groups (Fig. 2a). LPS administration at ZT14 elicited a decrease in food intake, which was significant on day 3 (*p <* 0.001) and day 5 (*p <* 0.001) post-injection without any interaction with ALAN (Fig. 2b). Changes in food intake were not detected in saline-injected groups at ZT2 (Fig. 2a), though a transient decrease was observed in rats injected with saline at ZT14 (Fig. 2b).

LPS-induced anorectic behaviour was accompanied by body weight loss (Fig. 2c,d). Administration of LPS at ZT2 caused a significant reduction of body weight in both CTRL and ALAN-exposed rats on post-treatment days 3 and 5 (*p <* 0.001; Fig. 2c). However, on day 3, the percentage change in body weight was significantly smaller in ALAN-exposed rats than in controls (*p <* 0.05; Fig. 2e). Under ALAN, body weight recovery was observed on day 8 post-LPS injection at ZT2, but the body weight of CTRL animals was still lower than the mean pre-treatment value (*p <* 0.001). LPS challenge at ZT14 reduced body weight 3, 5 and 8 days post-LPS (*p <* 0.001), without any differences between CTRL and ALAN-exposed rats (Fig. 2d,f). Together, the results indicate that dim ALAN attenuated the anorectic response when rats were exposed to the endotoxin during their early light phase.

### ALAN eliminates the time-of-day-dependent effect of LPS on the TNF-α and corticosterone response

**Figure 3:**
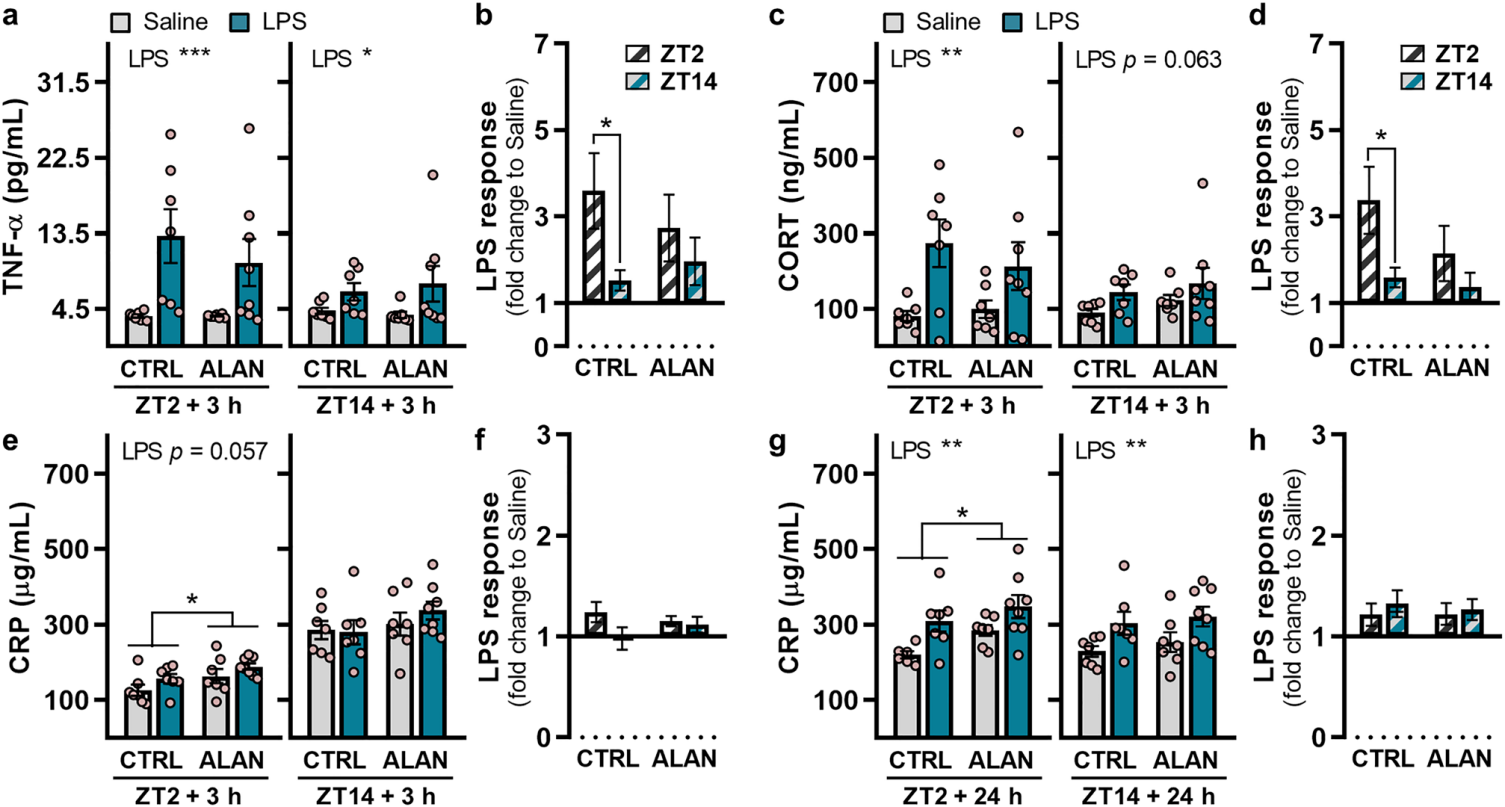
ALAN eliminates the time-of-day-dependent effect of lipopolysaccharide (LPS) on the tumour necrosis factor-α (TNF-α) and corticosterone (CORT) response. Absolute plasma/serum levels and the calculated LPS response normalized to the saline group for (**a**, **b**) TNF-α, (**c**, **d**) CORT and (**e–h**) C-reactive protein (CRP). Rats were exposed to either the control light/dark regime (CTRL) or ALAN (~2 lx) and injected with saline or LPS at either Zeitgeber time (ZT) 2 or ZT14. ZT0 = lights on. Blood samples were collected 3 and 24 h post-injection. Bars represent the mean ± SEM (n = 7–8 rats per group). Data for absolute levels were evaluated by two-way ANOVA. The LPS response between ZT2 and ZT14 was compared by the Student’s *t*-test. *p < 0.05, **p < 0.01 and ***p < 0.001.

Plasma melatonin levels were significantly lower during nights with dim light than during completely dark nights (*p <* 0.05; Supplementary Fig. S1). This is in line with our previous results, showing suppressed circadian melatonin oscillations under dim ALAN^11^. In both regimes, melatonin was not affected by LPS administration (Supplementary Fig. S1). TNF-α and CORT levels increased 3 h post-LPS challenge at ZT2 (*p <* 0.001 for TNF-α and *p <* 0.01 for CORT; Fig. 3a,c). LPS administration at ZT14 induced weaker response for TNF-α (*p <* 0.05; Fig. 3a) and non-significant increase for CORT (*p =* 0.063; Fig. 3c). ALAN-exposed rats also showed elevated TNF-α and CORT levels in response to LPS, but day-night variability observed in CTRL rats (*p <* 0.05) was missing under the ALAN regime (Fig. 3b,d).

The increase in plasma CRP was borderline significant 3 h post-LPS stimulation at ZT2 (*p =* 0.057; Fig. 3e) and was significant 24 h post-LPS administration at both ZT2 and ZT14 (*p <* 0.01; Fig. 3g). There was no time-dependent variability in the CRP response to LPS (Fig. 3f,h). Interestingly, ALAN-exposed rats showed overall higher CRP levels than CTRL animals during the light phase (*p <* 0.05), whereas these group differences were not observed during the night (Fig. 3e,g). This indicates that ALAN disturbs daily CRP rhythm, which can participate in altered day-night inflammatory responsiveness of the immune system.

### ALAN disturbs the time-dependent reactivity of neutrophils to LPS

**Figure 4:**
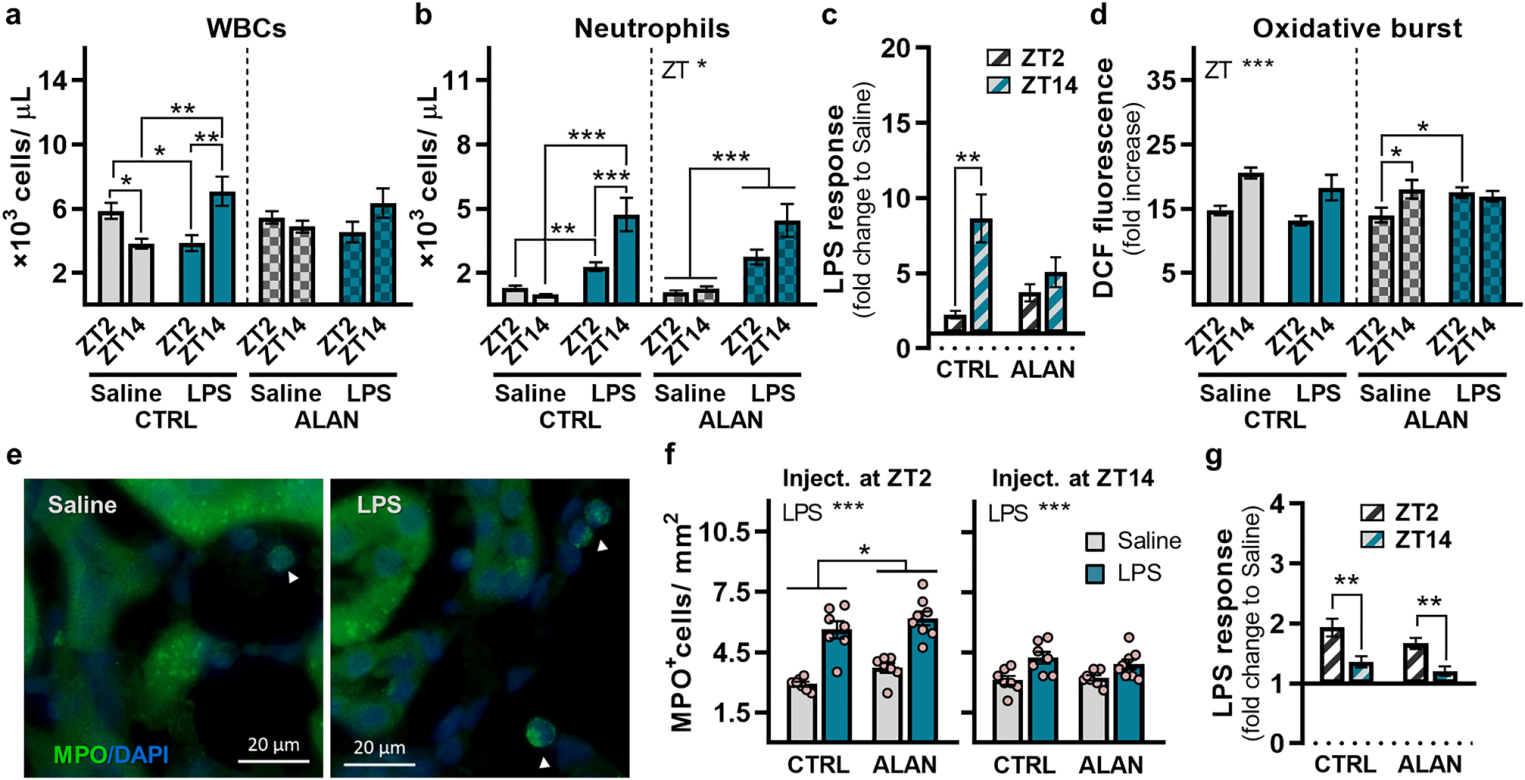
ALAN disturbs the time-dependent reactivity of neutrophils to lipopolysaccharide (LPS). Rats were exposed to either the control light/dark regime (CTRL) or ALAN (~2 lx) and injected with saline or LPS at either Zeitgeber time (ZT) 2 or ZT14. ZT0 = lights on. (**a**) Number of total white blood cells (WBCs), (**b**) number of blood neutrophils and (**c**) their calculated LPS response normalized to the saline group analysed 24 h post-injection. (**d**) The oxidative burst of blood neutrophils 24 h post-injection was calculated as the fold increase in the median dichlorofluorescein (DCF) fluorescence of the phorbol myristate acetate-stimulated over the non-stimulated neutrophils. (**e**) Representative immunofluorescent images of myeloperoxidase (MPO, green) counterstained with DAPI (blue) in the sections of the renal cortex 3 h post-injection. Arrowheads indicate MPO^+^ cells. (**f**) Quantification of MPO^+^ cells from immunofluorescent images and (**g**) the calculated LPS response. Bars represent the mean ± SEM (n = 7–8 rats per group). Data were evaluated by two-way ANOVA with Bonferroni’s multiple comparisons test. The LPS response between ZT2 and ZT14 was compared by Student’s *t*-test. *p < 0.05, **p < 0.01 and ***p < 0.001.

Neutrophils play an important role during acute inflammation and show rhythmic patterns in many aspects of their physiology, which are entrained by the L/D cycle^21^. Thus, we examined how blood neutrophil counts and their functional activity will change 24 h post-LPS challenge at either ZT2 or ZT14 and whether these responses will be affected by ALAN. In the CTRL regime, daily variability in total WBCs was inverted by time-of-day-dependent injection of LPS, since circulating counts were reduced following stimulation at ZT2 (*p <* 0.05) and elevated by stimulation at ZT14 (*p <* 0.01; Fig. 4a). This daily variability and response to LPS at the level of total WBCs was eliminated in ALAN-exposed rats (Fig. 4a), indicating that the proportional response of individual leukocyte types was changed. Peripheral neutrophilia was detected in both CTRL and ALAN-exposed rats (Fig. 4b). CTRL rats showed a more pronounced rise in neutrophil counts after LPS challenge at ZT14 compared with ZT2 (*p <* 0.01), but this time-dependent response was eliminated under the ALAN regime (Fig. 4c). Moreover, the daily pattern of neutrophil counts in saline-injected animals was altered in the ALAN group, as indicated by the lack of interaction between ZT and LPS (Fig. 4b).

The ability of neutrophils to produce ROS against pathogens is an important determinant of their killing function^20^. In the CTRL regime, the oxidative burst of neutrophils showed daily variability in both saline and LPS-treated animals (*p <* 0.001), while LPS did not exert a priming effect on the oxidative burst (Fig. 4d). Interestingly, in ALAN-exposed rats, LPS administered at ZT2 primed the oxidative burst (*p <* 0.05), thereby eliminating the daily variability observed in the saline-injected group (Fig. 4d). Basal ROS production was enhanced in neutrophils following LPS treatment, irrespective of the time of stimulation and lighting regime (Supplementary Fig. S2).

To evaluate neutrophil recruitment into the tissues, we quantified MPO-positive cells in kidney sections 3 h post-LPS challenge (Fig. 4e). LPS stimulation elevated the neutrophil numbers in both CTRL and ALAN-exposed animals (*p <* 0.001; Fig. 4f), with higher neutrophil infiltration in response to LPS administered at ZT2 compared with ZT14 (Fig. 4g). However, independent of LPS, ALAN-exposed rats had higher renal neutrophil counts than controls during the light phase (*p <* 0.05, Fig. 4f), a period when fewer neutrophils are present in the kidney compared with the dark phase^21^. Together, our data show that in response to endotoxin, an elevation in circulating and renal neutrophils shows mutually inverse day-night differences, and this time-dependent pattern is impaired by ALAN. Moreover, ALAN perturbs the time-dependent capacity of neutrophils to produce an oxidative burst under inflammatory conditions, and all these effects of ALAN can result from a disturbed steady-state daily rhythm of neutrophil trafficking between blood and the kidney.

### Effects of ALAN on the inflammatory responses of blood monocytes and renal macrophages

**Figure 5:**
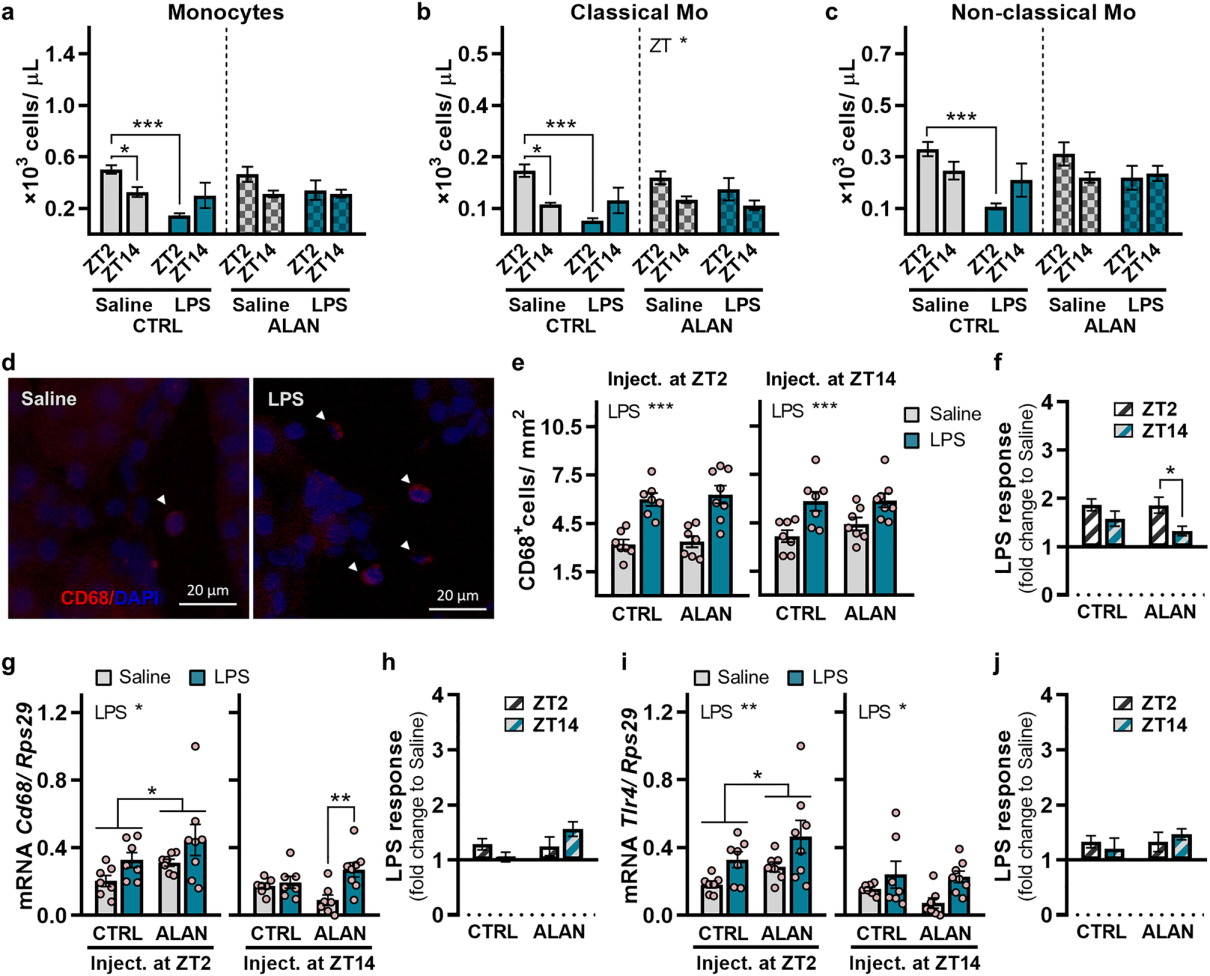
Effects of ALAN on the inflammatory responses of blood and renal monocytes (Mo)/macrophages. Rats were exposed to either the control light/dark regime (CTRL) or ALAN (~2 lx) and injected with saline or lipopolysaccharide (LPS) at either Zeitgeber time (ZT) 2 or ZT14. ZT0 = lights on. (**a**) The numbers of total blood monocytes, (**b**) classical CD43^lo^HIS48^hi^ and (**c**) non-classical CD43^hi^HIS48^lo^ monocyte subsets 24 h post-injection. (**d**) Representative immunofluorescent images of macrophage marker CD68 (red) counterstained with DAPI (blue) in the renal cortex 3 h post-injection. Arrowheads indicate CD68^+^ cells. (**e**) Quantification of CD68^+^ cells from immunofluorescent images and (**f**) the calculated LPS response normalized to the saline group. (**g**–**j**) Relative mRNA levels and the calculated LPS response for (**g**, **h**) *Cd68* and (**i**, **j**) Toll-like receptor 4 (*Tlr4*) in the renal cortex 3 h post-injection. Bars represent the mean ± SEM (n = 7–8 rats per group). Data were evaluated by two-way ANOVA with Bonferroni’s multiple comparisons test. The LPS response between ZT2 and ZT14 was compared by Student’s *t*-test. *p < 0.05, **p < 0.01 and ***p < 0.001.

Next, we measured the effects of ALAN on time-dependent changes in blood monocytes 24 h post-LPS stimulation. In CTRL rats, LPS challenge at ZT2 reduced total monocyte counts (*p <* 0.001), whereas no significant response was observed following LPS administration at ZT14 (Fig. 5a). Interestingly, in ALAN-exposed rats, total monocyte counts did not change in response to LPS at either ZT2 or ZT14. The same pattern was observed for subpopulations of classical (Fig. 5b) and non-classical monocytes (Fig. 5c).

Three hours post-LPS administration, the increased number of CD68-positive cells was found in the renal cortex of both CTRL and ALAN-exposed rats (*p <* 0.001 for LPS injection at ZT2 and ZT14; Fig. 5d,e). Day-night variability in this response was significant in ALAN (*p <* 0.05) but not in CTRL animals (Fig. 5f). Consistent with LPS-promoted macrophage infiltration, *Cd68* mRNA levels were up-regulated in the kidney upon LPS stimulation at ZT2 (*p <* 0.05; Fig. 5g). In response to LPS challenge at ZT14, *Cd68* mRNA levels did not change in CTRL animals, whereas a significant increase was observed in ALAN-exposed rats (*p <* 0.01; Fig. 5g,h). In the ALAN regime, rats showed higher daytime renal *Cd68* mRNA levels than controls (*p <* 0.05; Fig. 5g), suggesting a disturbed daily rhythm in this macrophage marker. ALAN and LPS-induced changes in renal *Tlr4* mRNA levels corresponded to *Cd68* expression (Fig. 5i,j), as *Tlr4* expression was daytime-specifically higher in ALAN-exposed rats than in controls (*p <* 0.05). Together, these results show that ALAN did not affect the inflammatory infiltration of macrophages into the kidney, although it enhanced the CD68-related inflammatory response upon night-time immune challenge.

### ALAN eliminates the time-dependent inflammatory response of blood lymphocytes

**Figure 6:**
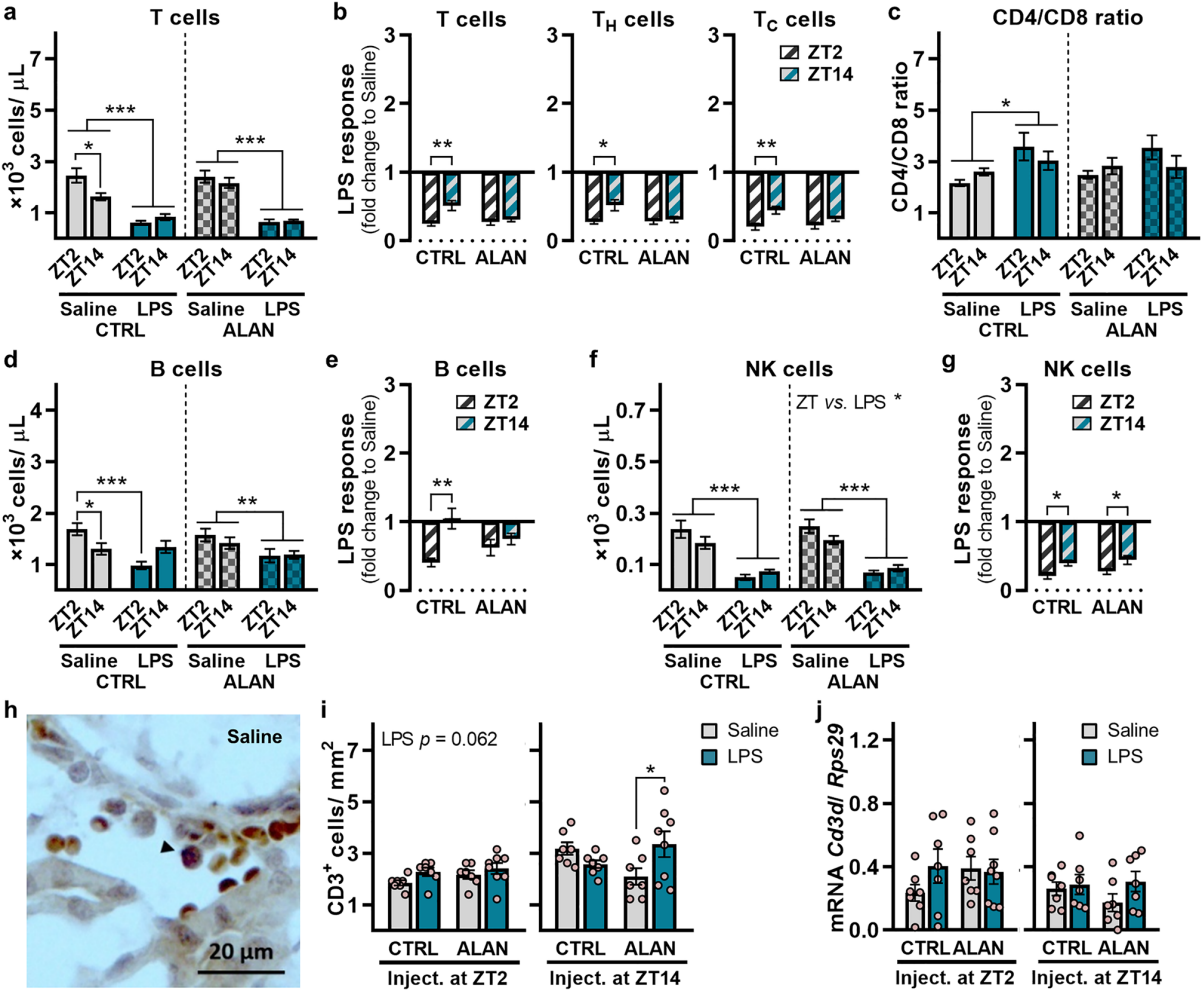
ALAN eliminates the time-of-day-dependent inflammatory response of blood lymphocytes. Rats were exposed to either the control light/dark regime (CTRL) or ALAN (~2 lx) and injected with saline or lipopolysaccharide (LPS) at either Zeitgeber time (ZT) 2 or ZT14. ZT0 = lights on. (**a**) The number of blood T cells, (**b**) the calculated LPS response normalized to the saline group for total T cells and T-cell subpopulations, (**c**) the CD4/CD8 ratio, (**d**, **e**) the absolute numbers and the calculated LPS response for B cells and (**f**, **g**) NK cells 24 h post-injection. (**h**) Immunohistochemical staining of CD3^+^ cells in the renal cortex 3 h post-injection. An arrowhead indicates CD3^+^ cell. (**i**) Quantification of CD3^+^ cells from immunohistochemical images. (**j**) Relative *Cd3d* mRNA levels in the renal cortex 3 h post-injection. Bars represent the mean ± SEM (n = 7–8 rats per group). Data were evaluated by two-way ANOVA with Bonferroni’s multiple comparisons test. The LPS response between ZT2 and ZT14 was compared by the Student’s *t*-test/Mann-Whitney test. *p < 0.05, **p < 0.01 and ***p < 0.001.

Next, we evaluated LPS-induced changes in lymphoid cells in the blood and recruitment of T cells into the renal cortex. The T-cell numbers displayed typical daily variability in the circulation of CTRL rats, showing higher numbers during the day than during the night (*p <* 0.05), but this daily pattern was suppressed under the ALAN regime (Fig. 6a). In both the CTRL and the ALAN groups, blood T cells decreased 24 h post-LPS challenge (*p <* 0.001; Fig. 6a). The reduction of T cells was enhanced upon LPS injection at ZT2 compared with ZT14 in controls (*p <* 0.01), whereas this time-dependent response was eliminated in ALAN-exposed rats (Fig. 6b). ALAN also abolished the time-dependent response to LPS in the blood counts of both helper T cells (T_H_) and cytotoxic T cells (T_C_) (Fig. 6b). Moreover, the CD4/CD8 ratio increased in response to LPS in CTRL rats (*p <* 0.05), while this LPS effect was not significant in the ALAN group (Fig. 6c). Like T cells, blood B-cell number varied across the day only under the CTRL regime (*p <* 0.05; Fig. 6d). In CTRL rats, B cells decreased following LPS injection at ZT2 (*p <* 0.001), while no response was observed upon LPS injection at ZT14 (Fig. 6d). The suppressive effect of LPS on B-cell number was not time-dependent in ALAN-exposed rats (Fig. 6e). LPS administration also reduced blood number of NK cells (*p <* 0.001; Fig. 6f) with significant day-night variability (*p <* 0.05), which was preserved under the ALAN regime (*p <* 0.05; Fig. 6g).

Renal infiltration with T cells was evaluated by the number of CD3-positive cells 3 h post-stimulation (Fig. 6h). LPS administered at ZT2 caused a non-significant increase of CD3-positive cells in the kidney independent of lighting conditions (*p =* 0.062), but when administered at ZT14, CD3-positive cells increased only in ALAN-exposed rats (*p <* 0.05; Fig. 6i). No differences were found for *Cd3* mRNA levels in the renal cortex (Fig. 6j). Thus, the data show that ALAN eliminated the time-of-day-dependent inflammatory response of blood lymphocytes and promoted inflammatory infiltration of T cells during the dim light phase.

### Effects of ALAN on NF-κB and molecules involved in renal inflammation

**Figure 7:**
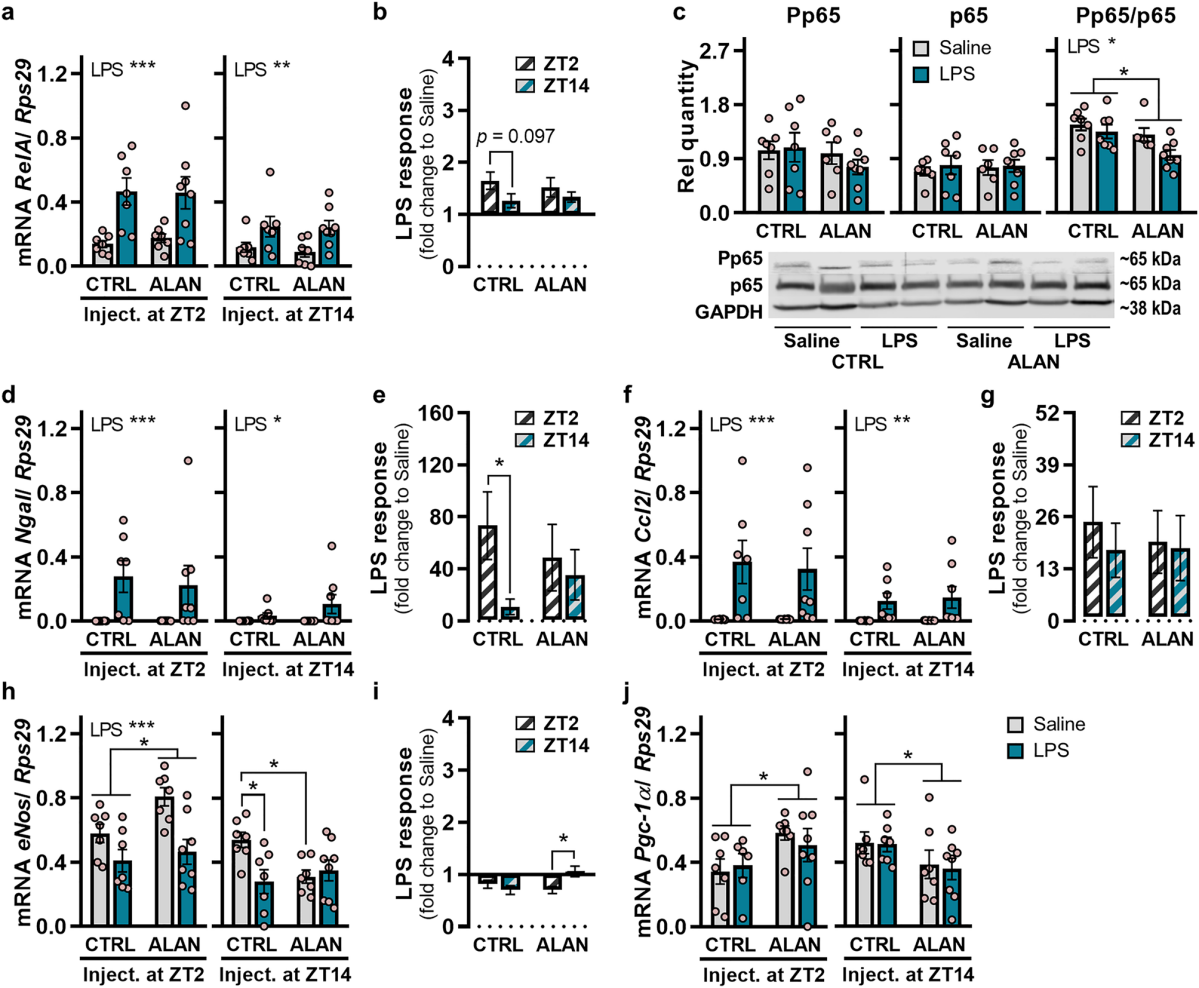
Effects of ALAN on the inflammatory response of NF-κB and immune-relevant genes in the kidney. Rats were exposed to either the control light/dark regime (CTRL) or ALAN (~2 lx) and injected with saline or lipopolysaccharide (LPS) at either Zeitgeber time (ZT) 2 or ZT14. ZT0 = lights on. The samples were collected 3 h post-injection. (**a**) *RelA* mRNA levels and (**b**) the calculated LPS response normalized to the saline group. (**c**) Protein levels of phosphorylated p65 (Pp65) and total p65 and the Pp65/p65 ratio post-injection at ZT2. (**d**–**j**) Relative mRNA levels and the calculated LPS response for (**d**, **e**) *Ngal*, (**f**, **g**) chemokine *Ccl2*, (**h**, **i**) endothelial nitric oxide synthase (*eNos*) and (**j**) transcriptional coactivator *Pgc-1α*. Bars represent the mean ± SEM (n = 7–8 rats per group). Data were evaluated by two-way ANOVA with Bonferroni’s multiple comparisons test. The LPS response between ZT2 and ZT14 was compared by the Student’s *t*-test/Mann-Whitney test. *p < 0.05, **p < 0.01 and ***p < 0.001.

In response to LPS, renal gene expression of NF-κB subunit *RelA* (*p65*) was up-regulated in both CTRL and ALAN-exposed rats (*p <* 0.001 for ZT2 and *p <* 0.01 for ZT14; Fig. 7a). In the CTRL regime, there was a non-significant trend towards a lower response post-LPS injection at ZT14 compared with ZT2 (*p =* 0.097), whereas no day-night variability was observed under ALAN (Fig. 7b). In the kidney, protein levels of phosphorylated p65 (Pp65) and total p65 were not affected by daytime LPS injection and lighting regime but the Pp65/p65 ratio was reduced by both ALAN (*p <* 0.05) and LPS stimulation (*p <* 0.05; Fig. 7c).

The NF-κB pathway controls the expression of numerous inflammatory genes, including NGAL, which is associated with renal pathologies and can further amplify the proinflammatory phenotype^22^. In the kidneys of both CTRL and ALAN-exposed rats, *Ngal* mRNA levels were up-regulated post-LPS injection (*p <* 0.001 for ZT2 and *p <* 0.05 for ZT14; Fig. 7d). CTRL animals showed lower *Ngal* up-regulation following LPS administration at ZT14 compared with ZT2 (*p <* 0.05), and this day-night variability was missing under the ALAN regime (Fig. 7e). However, no significant changes were found in protein NGAL levels post-LPS injection (Supplementary Fig. S3), which can be attributed to a delay between LPS-induced changes in the mRNA and protein levels^23^.

Additionally, we quantified the inflammatory response of specific chemokines, cell adhesion molecules and molecules that we previously identified to interfere with renal immune homeostasis under the ALAN regime^14^. LPS injection up-regulated renal mRNA levels of C-C motif chemokine ligand 2 (*Ccl2*) (*p <* 0.001 for ZT2 and *p <* 0.01 for ZT14; Fig. 7f,g), *Ccl5* (*p <* 0.05 for ZT2 and *p <* 0.01 for ZT14; Supplementary Fig. S4), and vascular cell adhesion molecule-1 (*Vcam1*) (*p <* 0.001 for ZT2 and ZT14; Supplementary Fig. S5), but this effect was not dependent on the time of LPS administration and the regime. Next, renal gene expression of endothelial nitric oxide synthase (*eNos*) was downregulated upon LPS injection at ZT2 regardless of lighting conditions (*p <* 0.001; Fig. 7h), but ALAN eliminated this response following LPS challenge at ZT14, thereby resulting in day-night differences in the LPS response (*p <* 0.05; Fig. 7i). This could be attributed to an altered daily pattern of *eNos* expression, as ALAN-exposed rats displayed higher *eNos* mRNA levels during the day (*p <* 0.05) and lower levels during the night, compared with controls (*p <* 0.05; Fig. 7h). The same ALAN-induced changes in the daily profile were detected for expression levels of a transcription coactivator, peroxisome proliferator-activated receptor gamma coactivator-1 alpha (*Pgc-1α*) (*p <* 0.05; Fig. 7j). Therefore, these findings could indicate that an altered daily profile of the renal genes is associated with disruption of local circadian regulation by the renal molecular clock.

### ALAN abolishes the acute Nr1d1 response to LPS in the kidney

**Figure 8:**
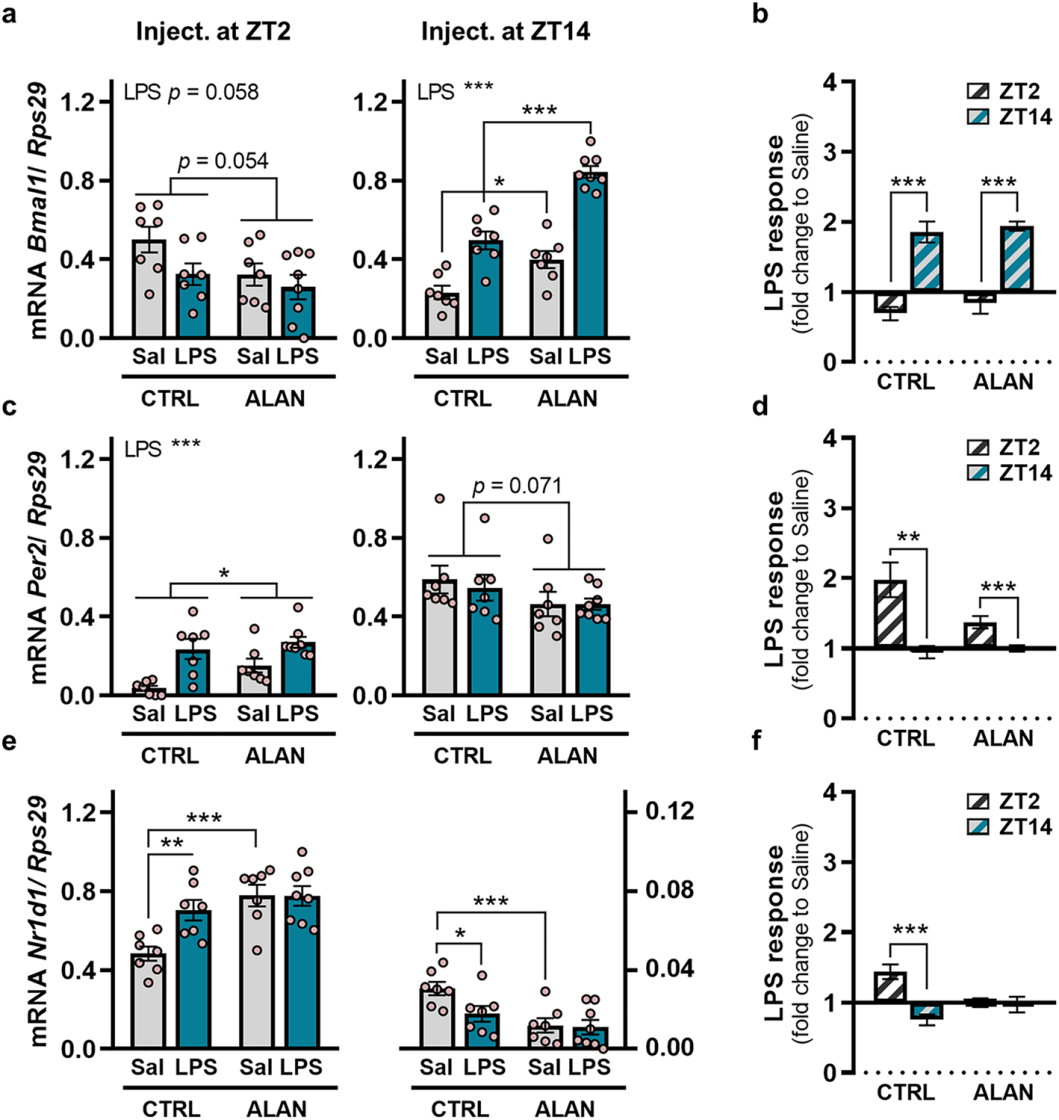
ALAN abolishes the acute *Nr1d1* response to LPS in the kidney. Rats were exposed to either the control light/dark regime (CTRL) or ALAN (~2 lx) and injected with saline (Sal) or lipopolysaccharide (LPS) at either Zeitgeber time (ZT) 2 or ZT14. ZT0 = lights on. Relative mRNA levels and the calculated LPS response normalized to the saline group for clock genes (**a**, **b**) *Bmal1*, (**c**, **d**) *Per2* and (**e**, **f**) *Nr1d1* analysed in the renal cortex 3 h post-injection. Bars represent the mean ± SEM (n = 7–8 rats per group). Data were evaluated by two-way ANOVA with Bonferroni’s multiple comparisons test. The LPS response was compared between ZT2 and ZT14 by the Student’s *t*-test/Mann–Whitney U test. *p < 0.05, **p < 0.01 and ***p < 0.001.

Next, we analysed whether ALAN could disturb the acute response to endotoxin at the level of the molecular clockwork in the kidney. Cellular molecular clocks consist of several clock genes (*Bmal1*, *Clock*, *Per1-3*, *Cry1/2*, *Nr1d1* and *Rorα*) and their protein products, which form interconnected transcriptional–translational feedback loops, driving rhythmic oscillations across the 24-h cycle^24^. The BMAL1/CLOCK heterodimer activates the rhythmic transcription of E-box-containing genes, such as *Per2* and *Nr1d1*, whereas PER/CRY proteins inhibit their own transcription. REV-ERBα is a repressive circadian regulator, which negatively controls *Bmal1* expression^25^. We found that *Bmal1* mRNA levels showed a borderline significant decrease following LPS administration at ZT2 (*p =* 0.058), but they increased significantly post-LPS challenge at ZT14 (*p <* 0.001; Fig. 8a), thereby showing a day-night variability, which was preserved in ALAN-exposed rats (*p <* 0.001; Fig. 8b). For *Per2* expression, a significant increase was induced by LPS administered at ZT2 (*p <* 0.001), and no response was found post-LPS challenge at ZT14 (Fig. 8c). ALAN did not eliminate this day-night variability (*p <* 0.001; Fig. 8d). In CTRL rats, renal *Nr1d1* expression increased following LPS challenge at ZT2 (*p <* 0.01) and, conversely, decreased post-LPS at ZT14 (*p <* 0.05; Fig. 8e). Abolishment of the *Nr1d1* response to LPS and elimination of day-night variability was observed in ALAN-exposed rats (Fig. 8f). Moreover, ALAN inversely changed renal expression of clock genes in the light and night phases, respectively (Fig. 8). Specifically, ALAN-exposed rats showed lower *Bmal1* mRNA and higher *Per2* and *Nr1d1* mRNA levels compared with CTRL rats during the light phase, while the reverse differences were found during the night phase (Fig. 8a,c,e). Altogether, under the standard L/D cycle, the response of renal clock genes to LPS showed daily variability and exposure to ALAN impaired such time-of-day-dependent inflammatory sensitivity of *Nr1d1* expression.

## Discussion

To analyse the time-of-day-dependent inflammatory response, rats were stimulated with LPS at either ZT2 or ZT14, which represent times with the maximum respectively minimum numbers of WBCs in the circulation^26^. Our data confirm daily variation for several components of the acute inflammatory response, meaning that the magnitude of changes in response to endotoxin varies with the timing of antigen exposure. Moreover, we demonstrated that compromising the standard L/D cycle with dim ALAN eliminated this time dependency at both the systemic and molecular levels. Dim ALAN suppressed daily oscillations of inflammatory changes in rat leukocyte numbers in the blood, including those of neutrophils, monocytes and lymphocytes, and in parallel, neutrophils displayed increased daytime sensitivity to the priming effects of LPS on oxidative burst. These effects appear to be consequent to disturbed homeostatic rhythms of leukocyte trafficking between the circulation and tissues, which was especially noted for neutrophil migration into the kidney. Dim ALAN also altered the renal inflammatory response, as was reflected in stimulated T-cell infiltration into the renal cortex upon night-time LPS challenge and a modified rhythmic response of genes involved in the inflammatory pathways, as well as in the renal molecular clock. We found impaired inflammatory responsiveness of *Nr1d1*, encoding REV-ERBα, and disrupted steady-state daily profile of the renal molecular clock under the ALAN regime, demonstrating a role of compromised circadian regulation in the chronodisruptive effects of ALAN on the acute inflammatory response.

ALAN-exposed rats, when administered LPS at the beginning of their light phase, responded with attenuated anorectic behaviour as compared with rats on the standard L/D regime. Experimental studies, employing SCN ablation, revealed that the central clock can significantly modulate the intensity of inflammatory responses^27^. In a recent paper, we demonstrated dampened rhythmicity of the molecular clockwork in the rat SCN caused by dim ALAN exposure, indicating compromised circadian timing function^11^. In addition, the study showed an altered daily pattern of food consumption in ALAN-exposed rats, which might also impact the magnitude of the anorectic response during immune challenge. In line with our current data, a reduced anorectic response to LPS at the beginning of the light phase was found in arrhythmic Siberian hamsters exposed to a disruptive phase-shifting protocol^28^. On the other hand, our recent data revealed that dim ALAN of 2 lx attenuated a day-night difference in locomotor activity but preserved its daily rhythmicity^12^. Anorectic behaviour is a component of sickness symptoms, which represent a set of adaptive responses to overcome infection^29^. It has been experimentally shown that fasting metabolism can be especially protective in bacterial inflammation, as blocking anorexia by glucose supplementation enhanced lethality in LPS-induced endotoxemia^30^. Thus, our data suggest that dim ALAN can weaken the adaptive anorectic response during bacterial inflammation.

Sickness symptoms, including anorexia, are controlled by inflammatory cytokines and hormones in the brain^29^. For example, CORT pre-treatment blocked the LPS-elicited development of anorexia in rats^31^. Here, we found an expected increase in TNF-α and CORT levels in the blood following LPS injection. Moreover, the LPS-induced rise in TNF-α and CORT was more pronounced in response to immune stimulation at the beginning of the rest than the active phase in rats on a control lighting regime. These observations agree with those of previous studies, which reported reduced responsiveness of the hypothalamic-pituitary adrenal (HPA) axis following endotoxin stimulation at the light-to-dark transition^32^. However, daily variation in CORT and TNF-α response to LPS was suppressed by ALAN. Recently, we showed that under steady-state conditions, dim ALAN resulted in an attenuated and advanced daily CORT rhythm^11^ and increased daytime CORT levels^14^. This indicates a role of disturbed CORT oscillations in altered responsiveness of the HPA axis during inflammation under dim ALAN conditions.

The inflammatory response involves dynamic changes in the number of leukocytes in the blood and tissues, and these changes depend on the timing of immune stimulation^33, 34^. In the CTRL regime, daytime and night-time LPS injection had inverse effects on the number of total WBCs. Neutrophils appear to be the main population contributing to this difference, as there was more pronounced neutrophilia upon immune challenge at ZT14 compared with ZT2. In ALAN-exposed rats, the daily oscillations in response to LPS were eliminated at the level of total WBCs, as well as that of neutrophils. Studies show that functional immune cell circadian clocks are critical for the manifestation of rhythmic neutrophil responses to parasitic or fungal infections^21, 35^. Therefore, it seems that ALAN-associated circadian disruption may impact the molecular clocks in neutrophils, compromising their inflammatory responses. Indeed, dim ALAN eliminated daily variability in the oxidative burst of blood neutrophils under inflammatory conditions. This likely resulted from the LPS-elicited priming of neutrophils, which was manifested specifically during the light phase in ALAN-exposed rats. Neutrophil priming can enhance the oxidative burst, though it must be tightly controlled to provide a benefit in terms of host defence and at the same time to avoid tissue damage from excessive priming^36^.

In our previous study, dim ALAN induced redox imbalance in the rat kidney under homeostatic conditions and we assumed that negative consequences of such imbalance could be expressed especially under inflammatory states^14^. In response to LPS, infiltration of WBCs into the renal cortex was increased, especially by neutrophils and macrophages, in both CTRL and ALAN-exposed rats. However, independent of immune stimulation, ALAN enhanced neutrophil migration into the kidney during the light phase, suggesting a disturbed daily rhythm. The migratory capacity of neutrophils is determined by their aging status in the circulation, which also varies over time and is controlled by the clock^21^. Importantly, these daily oscillations are supposed to balance the protective and destructive potential of neutrophils to optimize host defence to the time when the risk of infection is high^21^. Thus, if dim ALAN disturbs the circadian control of neutrophil rhythms, host defence mechanisms can be profoundly limited.

In contrast to neutrophils, monocytes and lymphocytes decline in the circulation during the first hours of acute inflammation^37^. Here, we observed decreased numbers of blood monocytes 24 h after daytime LPS stimulation and no changes following night-time LPS injection in the CTRL regime. It has been shown that the individual monocyte subsets exhibit differential kinetics in their loss and subsequent recovery in the circulation during the early stages of experimental endotoxemia^37^. Additionally, our results could indicate that monocyte kinetics during acute inflammation differ according to the time of antigen exposure. In ALAN-exposed rats, monocyte numbers were not affected 24 h post-LPS challenge regardless of the administration time, suggesting that dim ALAN impacts monocyte inflammatory kinetics. Next, we recorded decreased blood lymphocyte numbers induced by LPS in both CTRL and ALAN-exposed rats. In the CTRL regime, this decline was greater upon daytime than night-time LPS injection, but ALAN eliminated such day-night oscillations for T cells and B cells, as well as the main T-cell subsets. Circadian gating of inflammatory changes is particularly underlain by the steady-state daily rhythms of blood leukocytes^6^. Our current data revealed that dim ALAN disturbed steady-state daily variation of blood lymphocytes, whereas daily variation of monocytes was less affected. Nevertheless, this is consistent with our previous results, showing that monocytes lost their daily variability only after 5 weeks of dim ALAN exposure^14^.

Next, we observed that renal T-cell infiltration was stimulated upon LPS injection at ZT14 only in ALAN-exposed rats, indicating an enhanced inflammatory response during the active period. This was also supported by upregulation of renal *Cd68* only in rats under the ALAN following night-time LPS stimulation. CD68 is a lysosome-associated macrophage receptor and it is key to the antigen presentation^38^. Thus, our results may imply disturbed regulation of these immune processes during acute inflammation under dim ALAN. The effects of LPS are typically mediated by TLR4 signalling, which has also been shown to play a role in the circadian rhythmicity of macrophage inflammatory responses^39^. Here, the pattern of inflammatory changes in *Tlr4* expression was the same as that of *Cd68*. Moreover, renal *Cd68* and *Tlr4* expression levels were higher under the dim ALAN than the CTRL regime during the light phase, corresponding to our previous data^14^ and indicating compromised daily rhythms in the activation state of resident phagocytes in the kidney.

The timing of endotoxin administration determines the magnitude of transcriptional activity of NF-κB, which peaks upon stimulation in the middle of the light phase in mice^40^. In line with these data, we observed a trend towards more intense renal up-regulation of *RelA* post-LPS challenge at ZT2 compared with ZT14 in the CTRL regime. This day-night difference was missing in ALAN-exposed animals. Moreover, we recorded reduced Pp65/p65 ratio in the renal cortex of these rats, suggesting participation of the NF-κB pathway in chronodisruption of the acute inflammatory response. Correspondingly, dim ALAN abolished inflammatory day-night oscillations of the TNF-α increase in the circulation and *Ngal* up-regulation in the kidney. Both TNF-α and NGAL represent downstream targets of NF-κB^41, 42^. Importantly, in a mouse model of *Salmonella* infection, the phase-of-day-dependent response in caecal *Ngal* expression required a functional circadian clock^43^. Previous studies have highlighted several molecular mechanisms for crosstalk between the core clock components and the NF-κB pathway^40^. In the current study, dim ALAN affected steady-state renal expression of the clock genes *Bmal1*, *Per2* and *Nr1d1* in a time-of-day-dependent manner. This can indicate phase shifts in their daily rhythms, since our previous results showed phase-advanced rhythms of *Bmal1*, *Per2* and *Nr1d1* in the liver of rats exposed to dim ALAN^11, 12^. Therefore, we can suggest that ALAN-associated disruption of the local molecular clock interferes with the function of the renal inflammatory NF-κB pathway, which can promote disturbed rhythmicity of the acute inflammatory response.

Compromised circadian rhythms in the kidney of ALAN-exposed rats were further documented by an altered daily pattern of renal *eNos* and *Pgc-1α* expression. Increased daytime transcription of these genes agrees with our previous study and confirms disturbed renal homeostasis due to dim ALAN exposure^14^. Endothelial NOS contributes significantly to NO production in the kidney, playing an important role in the control of renal perfusion^44^. Moreover, NO generated by eNOS has been shown to regulate mitochondrial biogenesis through cGMP-dependent activation of *Pgc-1α* expression^45^. Additionally, both eNOS and PGC-1α display daily oscillations and have been implicated in the regulation of the circadian clock^46, 47^, suggesting that they could represent key players in the integration of kidney function and the circadian system. Our data also show that renal *eNos* expression was downregulated in response to LPS, corresponding with the published literature^48^. Interestingly, this decline was suppressed upon night-time LPS stimulation in ALAN-exposed rats, further indicating disruption of their time-of-day-dependent inflammatory sensitivity.

The inflammatory response can also affect the molecular clocks^49^. Here, LPS altered expression of clock genes in the kidney and the timing of immune stimulation significantly determined the direction of this response. In CTRL rats, endotoxin challenge in the early light phase up-regulated renal *Per2* and *Nr1d1* expression and tended to downregulate *Bmal1*. A contrasting response was elicited by LPS administered in the early dark phase, as *Bmal1* was up-regulated, *Nr1d1* was downregulated and no change was recorded for *Per2*. In other studies, the suppressive effects of systemic inflammation were reported for *Per1* and *Per2* expression in the liver, though the response was dependent on the time of stimulation, as well as the time post-injection^50, 51^. Thus, we hypothesize that the pattern of clock gene response upon LPS stimulation can be driven by the phase of the daily rhythm at the time of stimulation. Supportive evidence can be seen under the dim ALAN regime, which altered steady-state daily oscillations of clock genes in the kidney and this was associated with an inhibition of the inflammatory response of *Nr1d1*. The transcription factor REV-ERBα is an important repressor regulator in the molecular clockwork, which confers circadian rhythmicity on many target genes, including those involved in immunity. For example, an absence REV-ERBα in *Nr1d1*^−^/^−^ mice was manifested by missing circadian rhythmicity in LPS-induced IL6 response^5^ or increased severity of experimental colitis^52^.

## Conclusion

Our data demonstrate that compromised circadian timing function due to dim ALAN eliminates or modifies time-of-day-dependent oscillations of systemic and renal inflammatory responses. This may result from disturbed daily rhythms in leukocyte trafficking between the circulation and kidney and impaired inflammatory responsiveness of the renal molecular clock. The day-night imbalance in the sensitivity of immune defence mechanisms can point out an underlying link between light pollution and negative health effects.

### Supplementary Information

Supplementary Information.

## Data Availability

Data are available from the corresponding author upon reasonable request.
