# Supplementary material for: Chronodisruption of the acute inflammatory response by night lighting in rats
Source: Sci Rep. 2023 Aug 29;13:14109. doi: 10.1038/s41598-023-41266-3 (PMC10465576; doi:10.1038/s41598-023-41266-3)
Supplement: Supplementary file 1 — Supplementary Information. [file 41598_2023_41266_MOESM1_ESM.pdf]

Chronodisruption of the acute inflammatory response by night lighting in rats

Viera Jerigova, Michal Zeman, Monika Okuliarova

Supplementary Information

Supplementary Table S1. List of primary antibodies.

Gene	Method	Cat #	Source
FITC Anti-Rat CD3 (clone G4.18)	FC	11-0030	eBioscience
APC Anti-Rat CD4 (clone OX-35)	FC	17-0040	eBioscience
PE Anti-Rat CD8a (clone OX-8)	FC	12-0084	eBioscience
FITC Anti-Rat Granulocyte Marker (clone HIS48)	FC	11-0570	eBioscience
PE Anti-Rat CD45RA (clone OX-33)	FC	MR6404	Invitrogen
APC Anti-Rat CD161a (clone 10/78)	FC	MR6805	Invitrogen
PE-Cy7 Anti-Rat CD45 (clone OX-1)	FC	202214	BioLegend
PE Anti-Rat CD43 (clone W3/13)	FC	1614060	Sony Biotechnology
Mouse Anti-Rat CD68	IF	MCA341R	Bio-Rad
Rabbit Anti-Myeloperoxidase	IF	Ab9535	Abcam
Rabbit Anti-CD3	IH	Ab16669	Abcam
Rabbit Anti-Lipocalin-2 /NGAL	WB	Ab63929	Abcam
Rabbit Anti-NF-κB/p65 (phospho S536)	WB	Ab76302	Abcam
Rabbit Anti-NF-κB/p65	WB	Ab16502	Abcam
Mouse Anti-Glyceraldehyde-3-Phosphate Dehydrogenase	WB	MAB374	Sigma-Aldrich

Abbreviations: APC, allophycocyanin; FC, flow cytometry; FITC, fluorescein isothiocyanate; IF, immunofluorescence; IH, immunohistochemistry; PE, phycoerythrin; PE-Cy7, phycoerythrin-cyanine 7; WB, Western blot

Supplementary Table S2. Primer sequences for real-time PCR.

Gene	Accession Number	Forward primer	Reverse primer
Bmal1	NM_024362.2	5’-CACCTTGCGGAATGTCACAG-3’	5’-TACTTCCTTGGTCCACGGGT-3’
Ccl2	NM_031530.1	5’-TGTCTCAGCCAGATGCAGTTAAT-3’	5’-AGTTCTCCAGCCGACTCATTG-3’
Ccl5	NM_031116.3	5’-ACCTTGCAGTCGTCTTTGTC-3’	5’-TCTTGAACCCACTTCTTCTCTGG-3’
Cd3d	NM_013169.1	5’-CTCATCTTGGGCAAAGGCATC-3’	5’-ACAGTTCTGGCACATTCGGTAA-3’
Cd68	NM_001031638.1	5’-CGCATCTTGTACCTGACCCA-3’	5’-GCTCTGATGTCGGTCCTGTTT-3’
eNos	NM_021838.2	5’-TAACTCGATCAAAAGGAGTGGT-3’	5’-GCAGTCCCGAGCATCAAAT-3’
Ngal	NM_130741.1	5’-ATGCGGTCCAGAAAGAAAGAC-3’	5’-CCCTGACGAGGATGGAAGTG-3’
Nr1d1	NM_145775.2	5’-TCCCACATACTTCCCACCATCA-3’	5’-CACTCGGCTGCTGTCTTCCAT-3’
Per2	NM_031678.1	5’-TGGCAACCTTGAAGTACGCT-3’	5’-GCTGGCTCTCACTGGACATT-3’
Pgc-1α	NM_031347.1	5’-AACGATGACCCTCCTCACAC-3’	5’-GTTGTTGGTTTGGCTTGAGCA-3’
RelA	NM_199267.2	5’-ATTGTGTTCCGAACTCCCCC-3’	5’-TTTCTTCAATCCGGTGGCGA-3’
Rps29	NM_012876.1	5’-GCTGAACATGTGCCGACAGT-3’	5’-GGTCGCTTAGTCCAACTTAATGAA-3’
Tlr4	NM_019178.1	5’-TCACAACTTCAGTGGCTGGAT-3’	5’-TTGTCTCCACAGCCACCAGAT-3’
Vcam1	NM_012889.1	5’-GGAGTGAATCTGGTTGGGAGA-3’	5’-CAGCACATGTCAGAACAACGG-3’

Abbreviations: *Bmal1*, brain and muscle Arnt-like protein-1; *Ccl2*, C-C motif chemokine ligand 2; *Ccl5*, C-C motif chemokine ligand 5; *Cd3d*, CD3d molecule; *Cd68*, lysosome-associated macrophage receptor; *eNos*/*Nos3*, endothelial nitric oxide synthase; *Ngal*, lipocalin 2; *Nr1d1*, nuclear receptor subfamily 1, group D, member 1; *Per2*, period circadian regulator 2; *Pgc-1α*, peroxisome proliferator-activated receptor gamma coactivator-1 alpha; *RelA*, NF-kB subunit (p65); *Rps29*, ribosomal protein S29; *Tlr4*, toll-like receptor 4; *Vcam1*, vascular cell adhesion molecule-1.

Supplementary Figure S1

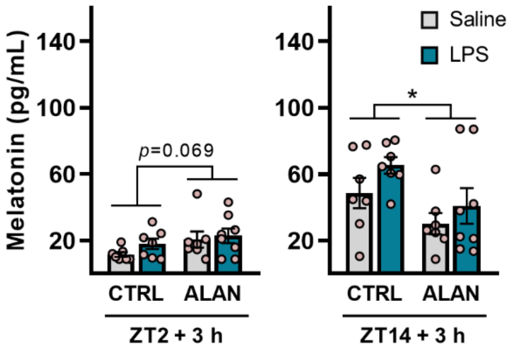

Figure S1. Artificial light at night (ALAN) supresses nocturnal melatonin levels. Rats were exposed to either the control light/dark regime (CTRL) or ALAN (~2 lx) and injected with saline or lipopolysaccharide (LPS) at either Zeitgeber time (ZT) 2 or ZT14. ZT0 = lights on. Blood samples were collected 3 h post-injection. Bars represent the mean ± SEM (n = 7–8 rats per group). Data were evaluated by two-way ANOVA. **p* < 0.05 for comparisons CTRL vs. ALAN groups.

Supplementary Figure S2

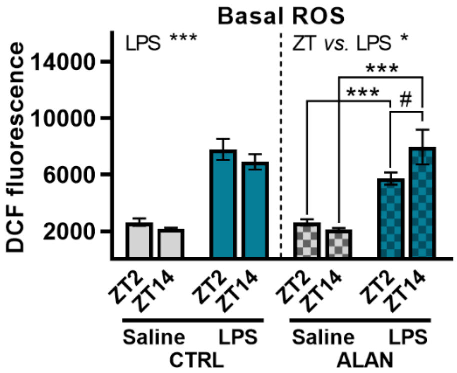

Figure S2. The effects of artificial light at night (ALAN) on steady-state and inflammatory production of reactive oxygen species (ROS) in blood neutrophils. Neutrophils were collected 24 h post-saline or lipopolysaccharide (LPS) injection at either Zeitgeber time (ZT) 2 or ZT14 from rats that were exposed to either the control light/dark regime (CTRL) or ALAN (~2 lx). ZT0 = lights on. The dichlorofluorescein (DCF) fluorescence is directly proportional to the ROS levels. Bars represent the mean ± SEM (n = 7–8 rats per group). Data were evaluated by two-way ANOVA with Bonferroni's multiple comparisons test. **p* < 0.05, ****p* < 0.001 and $^{\#}p$ = 0.075.

Supplementary Figure S3

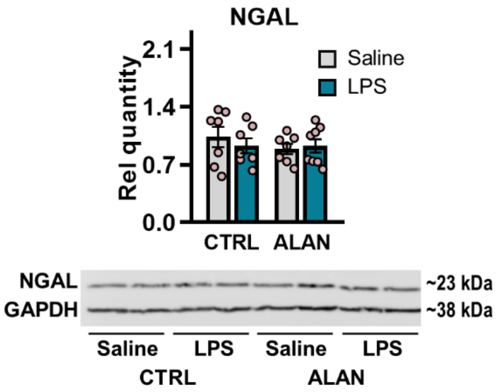

Figure S3. Effects of artificial light at night (ALAN) on the inflammatory response of lipocalin 2 (NGAL) protein levels in the kidney. Rats were exposed to either the control light/dark regime (CTRL) or ALAN (~2 lx) and sampled 3 h post-injection with saline or lipopolysaccharide (LPS) at Zeitgeber time (ZT) 2. ZT0 = lights on. NGAL protein levels were normalized to glyceraldehyde 3-phosphate dehydrogenase (GAPDH). Bars represent the mean ± SEM (n = 7–8 rats per group). Data were evaluated by two-way ANOVA.

Supplementary Figure S4

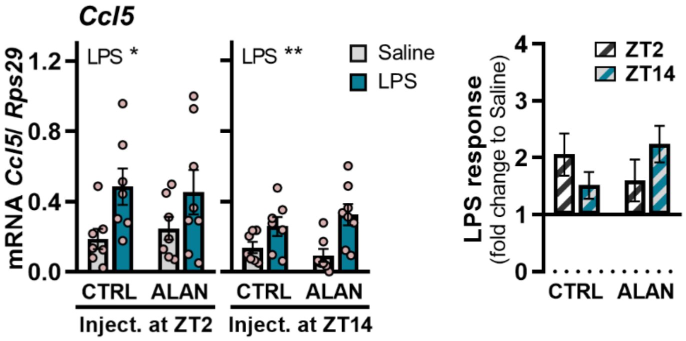

Figure S4. Effects of artificial light at night (ALAN) on the inflammatory response of chemokine CCL5 in the kidney. Relative *Ccl5* mRNA levels and the calculated LPS response normalized to the saline group in rats that were exposed to either the control light/dark regime (CTRL) or ALAN (~2 lx) and sampled 3 h post-injection with saline or lipopolysaccharide (LPS) at either Zeitgeber time (ZT) 2 or ZT14. ZT0 = lights on. Bars represent the mean ± SEM (n = 7–8 rats per group). Data were evaluated by two-way ANOVA at *$p < 0.05$ and **$p < 0.01$. The LPS response was compared between ZT2 and ZT14 by the Student's *t*-test.

Supplementary Figure S5

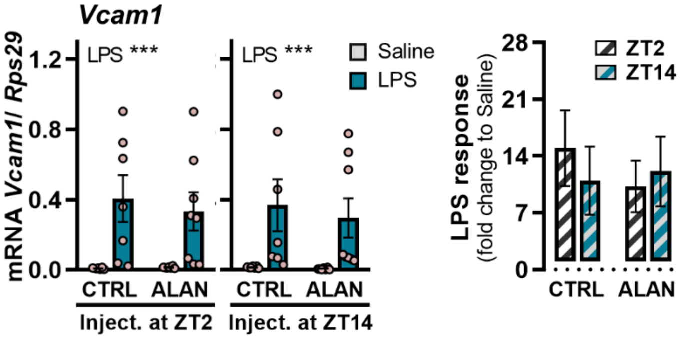

Figure S5. Effects of artificial light at night (ALAN) on the inflammatory response of vascular cell adhesion molecule-1 (VCAM1) in the kidney. Relative *Vcam1* mRNA levels and the calculated LPS response normalized to the saline group in rats that were exposed to either the control light/dark regime (CTRL) or ALAN (~2 lx) and sampled 3 h post-injection with saline or lipopolysaccharide (LPS) at either Zeitgeber time (ZT) 2 or ZT14. ZT0 = lights on. Bars represent the mean ± SEM (n = 7–8 rats per group). Data were evaluated by two-way ANOVA at ***$p < 0.001$. The LPS response was compared between ZT2 and ZT14 by the Student's *t*-test/Mann–Whitney test.
